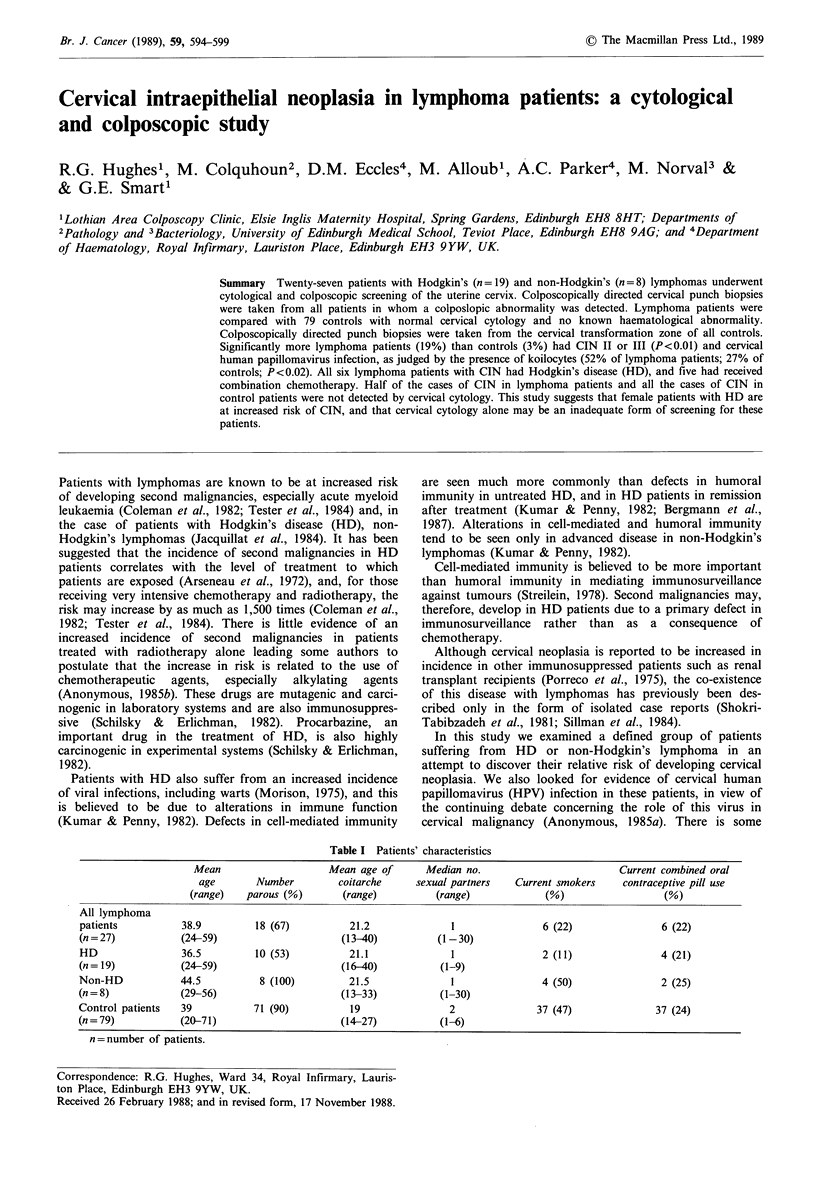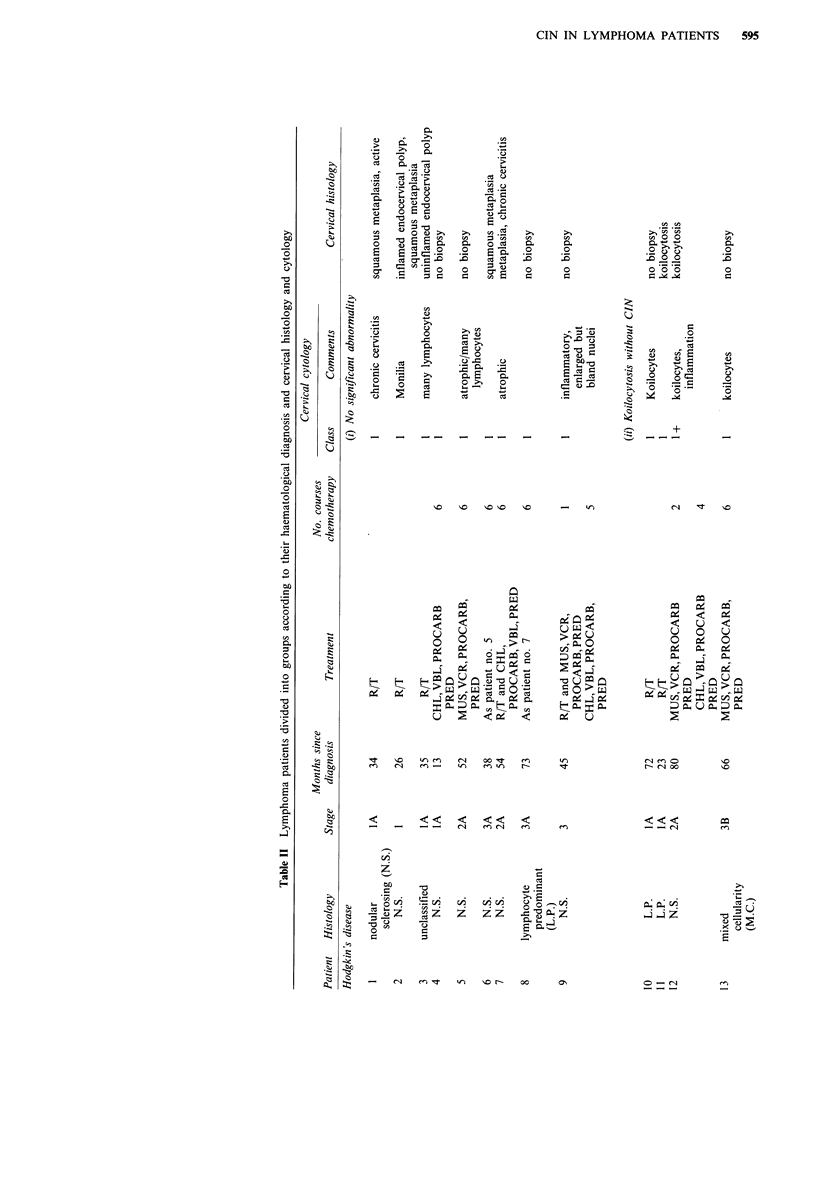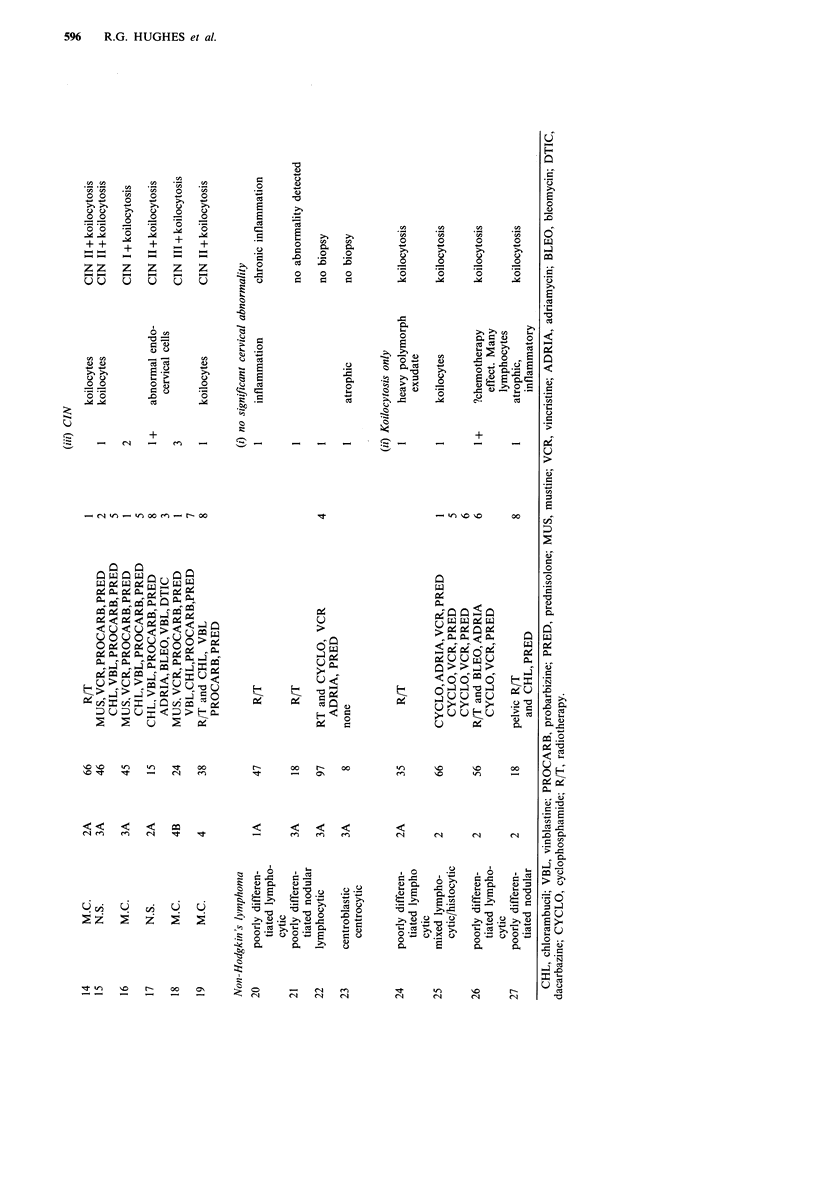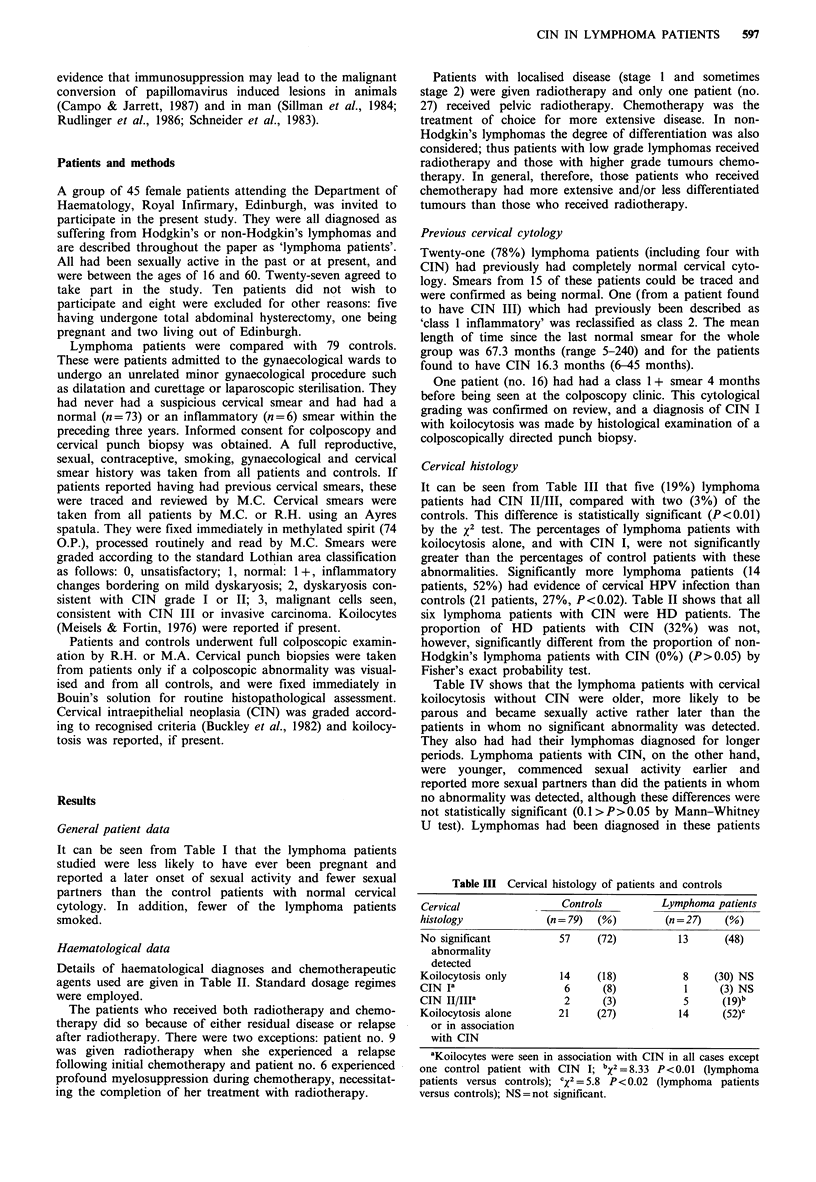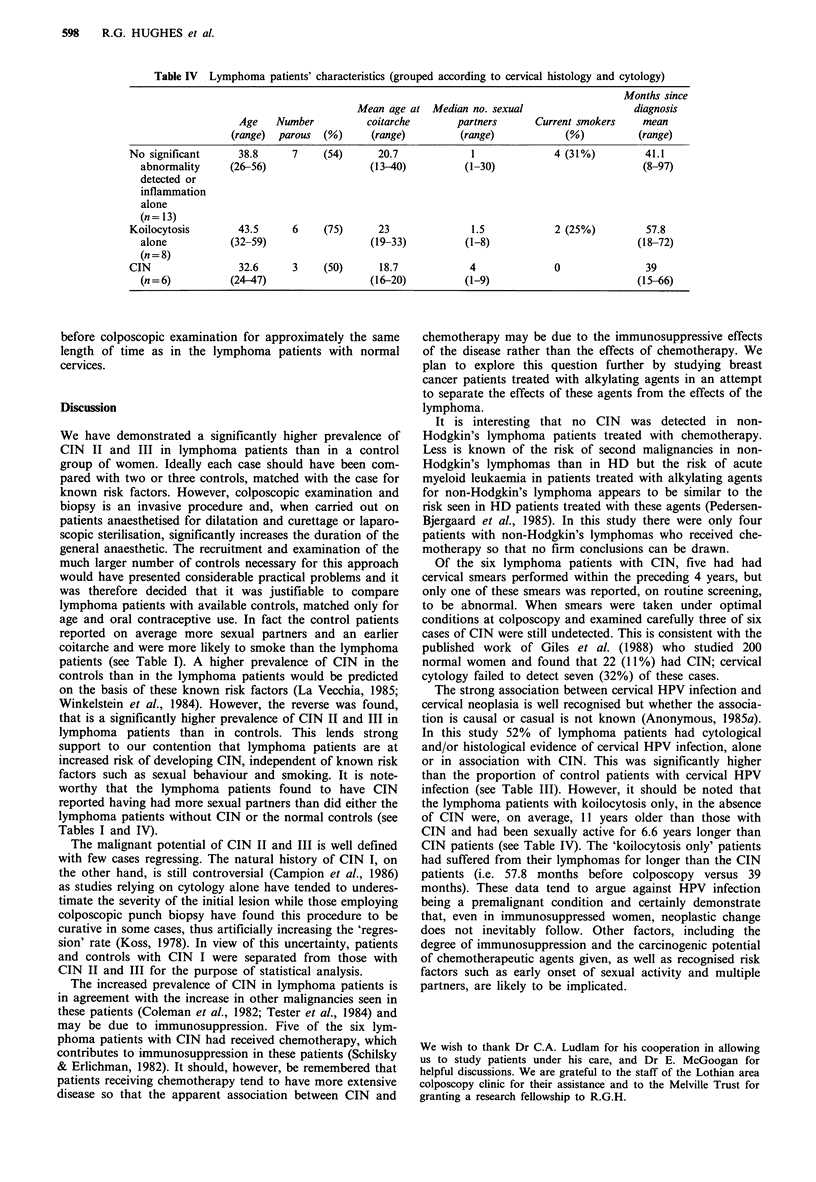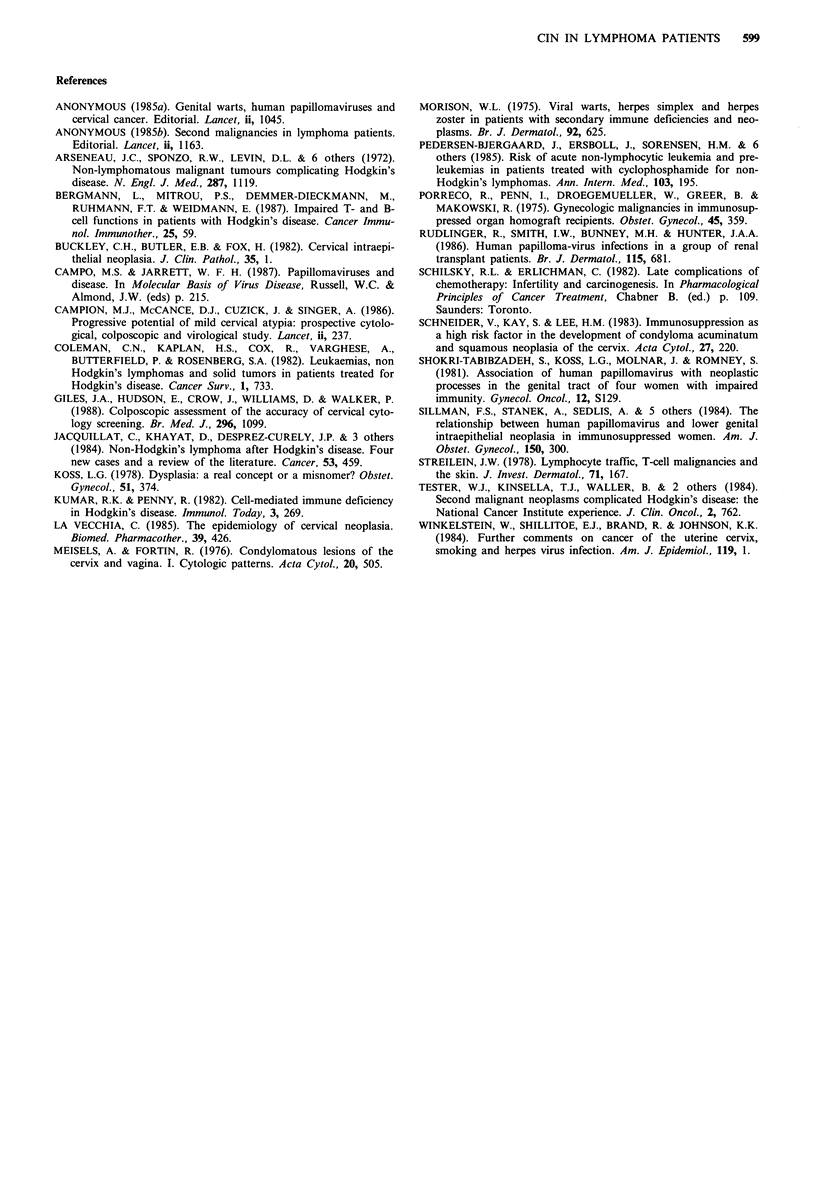# Cervical intraepithelial neoplasia in lymphoma patients: a cytological and colposcopic study.

**DOI:** 10.1038/bjc.1989.120

**Published:** 1989-04

**Authors:** R. G. Hughes, M. Colquhoun, D. M. Eccles, M. Alloub, A. C. Parker, M. Norval, G. E. Smart

**Affiliations:** Lothian Area Colposcopy Clinic, Elsie Inglis Maternity Hospital, Edinburgh, UK.

## Abstract

Twenty-seven patients with Hodgkin's (n = 19) and non-Hodgkin's (n = 8) lymphomas underwent cytological and colposcopic screening of the uterine cervix. Colposcopically directed cervical punch biopsies were taken from all patients in whom a colposcopic abnormality was detected. Lymphoma patients were compared with 79 controls with normal cervical cytology and no known haematological abnormality. Colposcopically directed punch biopsies were taken from the cervical transformation zone of all controls. Significantly more lymphoma patients (19%) than controls (3%) had CIN II or III (P less than 0.01) and cervical human papillomavirus infection, as judged by the presence of koilocytes (52% of lymphoma patients; 27% of controls; P less than 0.02). All six lymphoma patients with CIN had Hodgkin's disease (HD), and five had received combination chemotherapy. Half of the cases of CIN in lymphoma patients and all the cases of CIN in control patients were not detected by cervical cytology. This study suggests that female patients with HD are at increased risk of CIN, and that cervical cytology alone may be an inadequate form of screening for these patients.


Br. J. Cancer (1989), 59, 594–599 © The Macmillan Press Ltd., 1989

### Cervical intraepithelial neoplasia in lymphoma patients: a cytological and colposcopic study

R.G. Hughes[1], M. Colquhoun[2], D.M. Eccles[4], M. Alloub[1], A.C. Parker[4], M. Norval[3] & & G.E. Smart[1]

[1]*Lothian Area Colposcopy Clinic, Elsie Inglis Maternity Hospital, Spring Gardens, Edinburgh EH8 8HT; Departments of* [2]*Pathology and* [3]*Bacteriology, University of Edinburgh Medical School, Teviot Place, Edinburgh EH8 9AG; and* [4]*Department of Haematology, Royal Infirmary, Lauriston Place, Edinburgh EH3 9YW, UK.*

**Summary** Twenty-seven patients with Hodgkin's ($n=19$) and non-Hodgkin's ($n=8$) lymphomas underwent cytological and colposcopic screening of the uterine cervix. Colposcopically directed cervical punch biopsies were taken from all patients in whom a colposlopic abnormality was detected. Lymphoma patients were compared with 79 controls with normal cervical cytology and no known haematological abnormality. Colposcopically directed punch biopsies were taken from the cervical transformation zone of all controls. Significantly more lymphoma patients (19%) than controls (3%) had CIN II or III ($P<0.01$) and cervical human papillomavirus infection, as judged by the presence of koilocytes (52% of lymphoma patients; 27% of controls; $P<0.02$). All six lymphoma patients with CIN had Hodgkin's disease (HD), and five had received combination chemotherapy. Half of the cases of CIN in lymphoma patients and all the cases of CIN in control patients were not detected by cervical cytology. This study suggests that female patients with HD are at increased risk of CIN, and that cervical cytology alone may be an inadequate form of screening for these patients.

Patients with lymphomas are known to be at increased risk of developing second malignancies, especially acute myeloid leukaemia (Coleman *et al.*, 1982; Tester *et al.*, 1984) and, in the case of patients with Hodgkin's disease (HD), non-Hodgkin's lymphomas (Jacquillat *et al.*, 1984). It has been suggested that the incidence of second malignancies in HD patients correlates with the level of treatment to which patients are exposed (Arseneau *et al.*, 1972), and, for those receiving very intensive chemotherapy and radiotherapy, the risk may increase by as much as 1,500 times (Coleman *et al.*, 1982; Tester *et al.*, 1984). There is little evidence of an increased incidence of second malignancies in patients treated with radiotherapy alone leading some authors to postulate that the increase in risk is related to the use of chemotherapeutic agents, especially alkylating agents (Anonymous, 1985*b*). These drugs are mutagenic and carcinogenic in laboratory systems and are also immunosuppressive (Schilsky & Erlichman, 1982). Procarbazine, an important drug in the treatment of HD, is also highly carcinogenic in experimental systems (Schilsky & Erlichman, 1982).

Patients with HD also suffer from an increased incidence of viral infections, including warts (Morison, 1975), and this is believed to be due to alterations in immune function (Kumar & Penny, 1982). Defects in cell-mediated immunity are seen much more commonly than defects in humoral immunity in untreated HD, and in HD patients in remission after treatment (Kumar & Penny, 1982; Bergmann *et al.*, 1987). Alterations in cell-mediated and humoral immunity tend to be seen only in advanced disease in non-Hodgkin's lymphomas (Kumar & Penny, 1982).

Cell-mediated immunity is believed to be more important than humoral immunity in mediating immunosurveillance against tumours (Streilein, 1978). Second malignancies may, therefore, develop in HD patients due to a primary defect in immunosurveillance rather than as a consequence of chemotherapy.

Although cervical neoplasia is reported to be increased in incidence in other immunosuppressed patients such as renal transplant recipients (Porreco *et al.*, 1975), the co-existence of this disease with lymphomas has previously been described only in the form of isolated case reports (Shokri-Tabibzadeh *et al.*, 1981; Sillman *et al.*, 1984).

In this study we examined a defined group of patients suffering from HD or non-Hodgkin's lymphoma in an attempt to discover their relative risk of developing cervical neoplasia. We also looked for evidence of cervical human papillomavirus (HPV) infection in these patients, in view of the continuing debate concerning the role of this virus in cervical malignancy (Anonymous, 1985*a*). There is some

**Table I** Patients' characteristics

| | *Mean age (range)* | *Number parous (%)* | *Mean age of coitarche (range)* | *Median no. sexual partners (range)* | *Current smokers (%)* | *Current combined oral contraceptive pill use (%)* |
|---|---|---|---|---|---|---|
| All lymphoma patients ($n=27$) | 38.9 (24–59) | 18 (67) | 21.2 (13–40) | 1 (1–30) | 6 (22) | 6 (22) |
| HD ($n=19$) | 36.5 (24–59) | 10 (53) | 21.1 (16–40) | 1 (1–9) | 2 (11) | 4 (21) |
| Non-HD ($n=8$) | 44.5 (29–56) | 8 (100) | 21.5 (13–33) | 1 (1–30) | 4 (50) | 2 (25) |
| Control patients ($n=79$) | 39 (20–71) | 71 (90) | 19 (14–27) | 2 (1–6) | 37 (47) | 37 (24) |

$n$ = number of patients.

Correspondence: R.G. Hughes, Ward 34, Royal Infirmary, Lauriston Place, Edinburgh EH3 9YW, UK.
Received 26 February 1988; and in revised form, 17 November 1988.

**Table II** Lymphoma patients divided into groups according to their haematological diagnosis and cervical histology and cytology

| Patient | Histology | Stage | Months since diagnosis | Treatment | No. courses chemotherapy | Cervical cytology Class | Cervical cytology Comments | Cervical histology |
|---|---|---|---|---|---|---|---|---|
| *Hodgkin's disease* | | | | | | *(i) No significant abnormality* | | |
| 1 | nodular sclerosing (N.S.) | 1A | 34 | R/T | | 1 | chronic cervicitis | squamous metaplasia, active |
| 2 | N.S. | 1 | 26 | R/T | | 1 | Monilia | inflamed endocervical polyp, squamous metaplasia |
| 3 | unclassified | 1A | 35 | R/T | | 1 | many lymphocytes | uninflamed endocervical polyp |
| 4 | N.S. | 1A | 13 | CHL, VBL, PROCARB PRED | 6 | 1 | | no biopsy |
| 5 | N.S. | 2A | 52 | MUS, VCR, PROCARB, PRED | 6 | 1 | atrophic/many lymphocytes | no biopsy |
| 6 | N.S. | 3A | 38 | As patient no. 5 | 6 | 1 | | squamous metaplasia |
| 7 | N.S. | 2A | 54 | R/T and CHL, PROCARB, VBL, PRED | 6 | 1 | atrophic | metaplasia, chronic cervicitis |
| 8 | lymphocyte predominant (L.P.) | 3A | 73 | As patient no. 7 | 6 | 1 | | no biopsy |
| 9 | N.S. | 3 | 45 | R/T and MUS, VCR, PROCARB, PRED<br>CHL, VBL, PROCARB, PRED | 1<br>5 | 1 | inflammatory, enlarged but bland nuclei | no biopsy |
| | | | | | | *(ii) Koilocytosis without CIN* | | |
| 10 | L.P. | 1A | 72 | R/T | | 1 | Koilocytes | no biopsy |
| 11 | L.P. | 1A | 23 | R/T | | 1 | | koilocytosis |
| 12 | N.S. | 2A | 80 | MUS, VCR, PROCARB PRED<br>CHL, VBL, PROCARB PRED | 2<br>4 | 1+ | koilocytes, inflammation | koilocytosis |
| 13 | mixed cellularity (M.C.) | 3B | 66 | MUS, VCR, PROCARB, PRED | 6 | 1 | koilocytes | no biopsy |

| | | | | | | | | |
|---|---|---|---|---|---|---|---|---|
| | | | | | | *(iii) CIN* | | |
| 14 | M.C. | 2A | 66 | R/T | 1 | | koilocytes | CIN II+koilocytosis |
| 15 | N.S. | 3A | 46 | MUS, VCR, PROCARB, PRED<br>CHL, VBL, PROCARB, PRED | 2<br>5 | 1 | koilocytes | CIN II+koilocytosis |
| 16 | M.C. | 3A | 45 | MUS, VCR, PROCARB, PRED<br>CHL, VBL, PROCARB, PRED | 1<br>5 | 2 | | CIN I+koilocytosis |
| 17 | N.S. | 2A | 15 | CHL, VBL, PROCARB, PRED<br>ADRIA, BLEO, VBL, DTIC | 8<br>3 | 1+ | abnormal endo-cervical cells | CIN II+koilocytosis |
| 18 | M.C. | 4B | 24 | MUS, VCR, PROCARB, PRED<br>VBL,CHL,PROCARB,PRED | 1<br>7 | 3 | | CIN III+koilocytosis |
| 19 | M.C. | 4 | 38 | R/T and CHL, VBL PROCARB, PRED | 8 | 1 | koilocytes | CIN II+koilocytosis |
| *Non-Hodgkin's lymphoma* | | | | | | *(i) no significant cervical abnormality* | | |
| 20 | poorly differen-tiated lympho-cytic | 1A | 47 | R/T | | 1 | inflammation | chronic inflammation |
| 21 | poorly differen-tiated nodular | 3A | 18 | R/T | | 1 | | no abnormality detected |
| 22 | lymphocytic | 3A | 97 | RT and CYCLO, VCR ADRIA, PRED | 4 | 1 | | no biopsy |
| 23 | centroblastic centrocytic | 3A | 8 | none | | 1 | atrophic | no biopsy |
| | | | | | | *(ii) Koilocytosis only* | | |
| 24 | poorly differen-tiated lympho cytic | 2A | 35 | R/T | | 1 | heavy polymorph exudate | koilocytosis |
| 25 | mixed lympho-cytic/histocytic | 2 | 66 | CYCLO, ADRIA, VCR, PRED<br>CYCLO, VCR, PRED<br>CYCLO, VCR, PRED | 1<br>5<br>6 | 1 | koilocytes | koilocytosis |
| 26 | poorly differen-tiated lympho-cytic | 2 | 56 | R/T and BLEO, ADRIA CYCLO, VCR, PRED | 6 | 1+ | ?chemotherapy effect. Many lymphocytes | koilocytosis |
| 27 | poorly differen-tiated nodular | 2 | 18 | pelvic R/T and CHL, PRED | 8 | 1 | atrophic, inflammatory | koilocytosis |

CHL, chlorambucil; VBL, vinblastine; PROCARB, probarbizine; PRED, prednisolone; MUS, mustine; VCR, vincristine; ADRIA, adriamycin; BLEO, bleomycin; DTIC, dacarbazine; CYCLO, cyclophosphamide; R/T, radiotherapy.

evidence that immunosuppression may lead to the malignant conversion of papillomavirus induced lesions in animals (Campo & Jarrett, 1987) and in man (Sillman *et al.*, 1984; Rudlinger *et al.*, 1986; Schneider *et al.*, 1983).

### Patients and methods

A group of 45 female patients attending the Department of Haematology, Royal Infirmary, Edinburgh, was invited to participate in the present study. They were all diagnosed as suffering from Hodgkin's or non-Hodgkin's lymphomas and are described throughout the paper as 'lymphoma patients'. All had been sexually active in the past or at present, and were between the ages of 16 and 60. Twenty-seven agreed to take part in the study. Ten patients did not wish to participate and eight were excluded for other reasons: five having undergone total abdominal hysterectomy, one being pregnant and two living out of Edinburgh.

Lymphoma patients were compared with 79 controls. These were patients admitted to the gynaecological wards to undergo an unrelated minor gynaecological procedure such as dilatation and curettage or laparoscopic sterilisation. They had never had a suspicious cervical smear and had had a normal ($n=73$) or an inflammatory ($n=6$) smear within the preceding three years. Informed consent for colposcopy and cervical punch biopsy was obtained. A full reproductive, sexual, contraceptive, smoking, gynaecological and cervical smear history was taken from all patients and controls. If patients reported having had previous cervical smears, these were traced and reviewed by M.C. Cervical smears were taken from all patients by M.C. or R.H. using an Ayres spatula. They were fixed immediately in methylated spirit (74 O.P.), processed routinely and read by M.C. Smears were graded according to the standard Lothian area classification as follows: 0, unsatisfactory; 1, normal: 1+, inflammatory changes bordering on mild dyskaryosis; 2, dyskaryosis consistent with CIN grade I or II; 3, malignant cells seen, consistent with CIN III or invasive carcinoma. Koilocytes (Meisels & Fortin, 1976) were reported if present.

Patients and controls underwent full colposcopic examination by R.H. or M.A. Cervical punch biopsies were taken from patients only if a colposcopic abnormality was visualised and from all controls, and were fixed immediately in Bouin's solution for routine histopathological assessment. Cervical intraepithelial neoplasia (CIN) was graded according to recognised criteria (Buckley *et al.*, 1982) and koilocytosis was reported, if present.

### Results

### *General patient data*

It can be seen from Table I that the lymphoma patients studied were less likely to have ever been pregnant and reported a later onset of sexual activity and fewer sexual partners than the control patients with normal cervical cytology. In addition, fewer of the lymphoma patients smoked.

### *Haematological data*

Details of haematological diagnoses and chemotherapeutic agents used are given in Table II. Standard dosage regimes were employed.

The patients who received both radiotherapy and chemotherapy did so because of either residual disease or relapse after radiotherapy. There were two exceptions: patient no. 9 was given radiotherapy when she experienced a relapse following initial chemotherapy and patient no. 6 experienced profound myelosuppression during chemotherapy, necessitating the completion of her treatment with radiotherapy.

Patients with localised disease (stage 1 and sometimes stage 2) were given radiotherapy and only one patient (no. 27) received pelvic radiotherapy. Chemotherapy was the treatment of choice for more extensive disease. In non-Hodgkin's lymphomas the degree of differentiation was also considered; thus patients with low grade lymphomas received radiotherapy and those with higher grade tumours chemotherapy. In general, therefore, those patients who received chemotherapy had more extensive and/or less differentiated tumours than those who received radiotherapy.

### *Previous cervical cytology*

Twenty-one (78%) lymphoma patients (including four with CIN) had previously had completely normal cervical cytology. Smears from 15 of these patients could be traced and were confirmed as being normal. One (from a patient found to have CIN III) which had previously been described as 'class 1 inflammatory' was reclassified as class 2. The mean length of time since the last normal smear for the whole group was 67.3 months (range 5–240) and for the patients found to have CIN 16.3 months (6–45 months).

One patient (no. 16) had had a class 1+ smear 4 months before being seen at the colposcopy clinic. This cytological grading was confirmed on review, and a diagnosis of CIN I with koilocytosis was made by histological examination of a colposcopically directed punch biopsy.

### *Cervical histology*

It can be seen from Table III that five (19%) lymphoma patients had CIN II/III, compared with two (3%) of the controls. This difference is statistically significant ($P<0.01$) by the $\chi^2$ test. The percentages of lymphoma patients with koilocytosis alone, and with CIN I, were not significantly greater than the percentages of control patients with these abnormalities. Significantly more lymphoma patients (14 patients, 52%) had evidence of cervical HPV infection than controls (21 patients, 27%, $P<0.02$). Table II shows that all six lymphoma patients with CIN were HD patients. The proportion of HD patients with CIN (32%) was not, however, significantly different from the proportion of non-Hodgkin's lymphoma patients with CIN (0%) ($P>0.05$) by Fisher's exact probability test.

Table IV shows that the lymphoma patients with cervical koilocytosis without CIN were older, more likely to be parous and became sexually active rather later than the patients in whom no significant abnormality was detected. They also had had their lymphomas diagnosed for longer periods. Lymphoma patients with CIN, on the other hand, were younger, commenced sexual activity earlier and reported more sexual partners than did the patients in whom no abnormality was detected, although these differences were not statistically significant ($0.1>P>0.05$ by Mann–Whitney U test). Lymphomas had been diagnosed in these patients

**Table III** Cervical histology of patients and controls

| *Cervical histology* | *Controls* (*n*=*79*) | (*%*) | *Lymphoma patients* (*n*=*27*) | (*%*) |
|---|---|---|---|---|
| No significant abnormality detected | 57 | (72) | 13 | (48) |
| Koilocytosis only | 14 | (18) | 8 | (30) NS |
| CIN I[a] | 6 | (8) | 1 | (3) NS |
| CIN II/III[a] | 2 | (3) | 5 | (19)[b] |
| Koilocytosis alone or in association with CIN | 21 | (27) | 14 | (52)[c] |

[a]Koilocytes were seen in association with CIN in all cases except one control patient with CIN I; [b]$\chi^2=8.33$ $P<0.01$ (lymphoma patients versus controls); [c]$\chi^2=5.8$ $P<0.02$ (lymphoma patients versus controls); NS = not significant.

**Table IV** Lymphoma patients' characteristics (grouped according to cervical histology and cytology)

| | *Age* (*range*) | *Number parous* | (%) | *Mean age at coitarche* (*range*) | *Median no. sexual partners* (*range*) | *Current smokers* (%) | *Months since diagnosis mean* (*range*) |
|---|---|---|---|---|---|---|---|
| No significant abnormality detected or inflammation alone (*n* = 13) | 38.8 (26–56) | 7 | (54) | 20.7 (13–40) | 1 (1–30) | 4 (31%) | 41.1 (8–97) |
| Koilocytosis alone (*n* = 8) | 43.5 (32–59) | 6 | (75) | 23 (19–33) | 1.5 (1–8) | 2 (25%) | 57.8 (18–72) |
| CIN (*n* = 6) | 32.6 (24–47) | 3 | (50) | 18.7 (16–20) | 4 (1–9) | 0 | 39 (15–66) |

before colposcopic examination for approximately the same length of time as in the lymphoma patients with normal cervices.

### Discussion

We have demonstrated a significantly higher prevalence of CIN II and III in lymphoma patients than in a control group of women. Ideally each case should have been compared with two or three controls, matched with the case for known risk factors. However, colposcopic examination and biopsy is an invasive procedure and, when carried out on patients anaesthetised for dilatation and curettage or laparoscopic sterilisation, significantly increases the duration of the general anaesthetic. The recruitment and examination of the much larger number of controls necessary for this approach would have presented considerable practical problems and it was therefore decided that it was justifiable to compare lymphoma patients with available controls, matched only for age and oral contraceptive use. In fact the control patients reported on average more sexual partners and an earlier coitarche and were more likely to smoke than the lymphoma patients (see Table I). A higher prevalence of CIN in the controls than in the lymphoma patients would be predicted on the basis of these known risk factors (La Vecchia, 1985; Winkelstein *et al.*, 1984). However, the reverse was found, that is a significantly higher prevalence of CIN II and III in lymphoma patients than in controls. This lends strong support to our contention that lymphoma patients are at increased risk of developing CIN, independent of known risk factors such as sexual behaviour and smoking. It is noteworthy that the lymphoma patients found to have CIN reported having had more sexual partners than did either the lymphoma patients without CIN or the normal controls (see Tables I and IV).

The malignant potential of CIN II and III is well defined with few cases regressing. The natural history of CIN I, on the other hand, is still controversial (Campion *et al.*, 1986) as studies relying on cytology alone have tended to underestimate the severity of the initial lesion while those employing colposcopic punch biopsy have found this procedure to be curative in some cases, thus artificially increasing the 'regression' rate (Koss, 1978). In view of this uncertainty, patients and controls with CIN I were separated from those with CIN II and III for the purpose of statistical analysis.

The increased prevalence of CIN in lymphoma patients is in agreement with the increase in other malignancies seen in these patients (Coleman *et al.*, 1982; Tester *et al.*, 1984) and may be due to immunosuppression. Five of the six lymphoma patients with CIN had received chemotherapy, which contributes to immunosuppression in these patients (Schilsky & Erlichman, 1982). It should, however, be remembered that patients receiving chemotherapy tend to have more extensive disease so that the apparent association between CIN and chemotherapy may be due to the immunosuppressive effects of the disease rather than the effects of chemotherapy. We plan to explore this question further by studying breast cancer patients treated with alkylating agents in an attempt to separate the effects of these agents from the effects of the lymphoma.

It is interesting that no CIN was detected in non-Hodgkin's lymphoma patients treated with chemotherapy. Less is known of the risk of second malignancies in non-Hodgkin's lymphomas than in HD but the risk of acute myeloid leukaemia in patients treated with alkylating agents for non-Hodgkin's lymphoma appears to be similar to the risk seen in HD patients treated with these agents (Pedersen-Bjergaard *et al.*, 1985). In this study there were only four patients with non-Hodgkin's lymphomas who received chemotherapy so that no firm conclusions can be drawn.

Of the six lymphoma patients with CIN, five had had cervical smears performed within the preceding 4 years, but only one of these smears was reported, on routine screening, to be abnormal. When smears were taken under optimal conditions at colposcopy and examined carefully three of six cases of CIN were still undetected. This is consistent with the published work of Giles *et al.* (1988) who studied 200 normal women and found that 22 (11%) had CIN; cervical cytology failed to detect seven (32%) of these cases.

The strong association between cervical HPV infection and cervical neoplasia is well recognised but whether the association is causal or casual is not known (Anonymous, 1985*a*). In this study 52% of lymphoma patients had cytological and/or histological evidence of cervical HPV infection, alone or in association with CIN. This was significantly higher than the proportion of control patients with cervical HPV infection (see Table III). However, it should be noted that the lymphoma patients with koilocytosis only, in the absence of CIN were, on average, 11 years older than those with CIN and had been sexually active for 6.6 years longer than CIN patients (see Table IV). The 'koilocytosis only' patients had suffered from their lymphomas for longer than the CIN patients (i.e. 57.8 months before colposcopy versus 39 months). These data tend to argue against HPV infection being a premalignant condition and certainly demonstrate that, even in immunosuppressed women, neoplastic change does not inevitably follow. Other factors, including the degree of immunosuppression and the carcinogenic potential of chemotherapeutic agents given, as well as recognised risk factors such as early onset of sexual activity and multiple partners, are likely to be implicated.

We wish to thank Dr C.A. Ludlam for his cooperation in allowing us to study patients under his care, and Dr E. McGoogan for helpful discussions. We are grateful to the staff of the Lothian area colposcopy clinic for their assistance and to the Melville Trust for granting a research fellowship to R.G.H.

### References

ANONYMOUS (1985*a*). Genital warts, human papillomaviruses and cervical cancer. Editorial. *Lancet*, **ii**, 1045.

ANONYMOUS (1985*b*). Second malignancies in lymphoma patients. Editorial. *Lancet*, **ii**, 1163.

ARSENEAU, J.C., SPONZO, R.W., LEVIN, D.L. & 6 others (1972). Non-lymphomatous malignant tumours complicating Hodgkin's disease. *N. Engl. J. Med.*, **287**, 1119.

BERGMANN, L., MITROU, P.S., DEMMER-DIECKMANN, M., RUHMANN, F.T. & WEIDMANN, E. (1987). Impaired T- and B-cell functions in patients with Hodgkin's disease. *Cancer Immunol. Immunother.*, **25**, 59.

BUCKLEY, C.H., BUTLER, E.B. & FOX, H. (1982). Cervical intraepithelial neoplasia. *J. Clin. Pathol.*, **35**, 1.

CAMPO, M.S. & JARRETT, W. F. H. (1987). Papillomaviruses and disease. In *Molecular Basis of Virus Disease*, Russell, W.C. & Almond, J.W. (eds) p. 215.

CAMPION, M.J., McCANCE, D.J., CUZICK, J. & SINGER, A. (1986). Progressive potential of mild cervical atypia: prospective cytological, colposcopic and virological study. *Lancet*, **ii**, 237.

COLEMAN, C.N., KAPLAN, H.S., COX, R., VARGHESE, A., BUTTERFIELD, P. & ROSENBERG, S.A. (1982). Leukaemias, non Hodgkin's lymphomas and solid tumors in patients treated for Hodgkin's disease. *Cancer Surv.*, **1**, 733.

GILES, J.A., HUDSON, E., CROW, J., WILLIAMS, D. & WALKER, P. (1988). Colposcopic assessment of the accuracy of cervical cytology screening. *Br. Med. J.*, **296**, 1099.

JACQUILLAT, C., KHAYAT, D., DESPREZ-CURELY, J.P. & 3 others (1984). Non-Hodgkin's lymphoma after Hodgkin's disease. Four new cases and a review of the literature. *Cancer*, **53**, 459.

KOSS, L.G. (1978). Dysplasia: a real concept or a misnomer? *Obstet. Gynecol.*, **51**, 374.

KUMAR, R.K. & PENNY, R. (1982). Cell-mediated immune deficiency in Hodgkin's disease. *Immunol. Today*, **3**, 269.

LA VECCHIA, C. (1985). The epidemiology of cervical neoplasia. *Biomed. Pharmacother.*, **39**, 426.

MEISELS, A. & FORTIN, R. (1976). Condylomatous lesions of the cervix and vagina. I. Cytologic patterns. *Acta Cytol.*, **20**, 505.

MORISON, W.L. (1975). Viral warts, herpes simplex and herpes zoster in patients with secondary immune deficiencies and neoplasms. *Br. J. Dermatol.*, **92**, 625.

PEDERSEN-BJERGAARD, J., ERSBOLL, J., SORENSEN, H.M. & 6 others (1985). Risk of acute non-lymphocytic leukemia and preleukemias in patients treated with cyclophosphamide for non-Hodgkin's lymphomas. *Ann. Intern. Med.*, **103**, 195.

PORRECO, R., PENN, I., DROEGEMUELLER, W., GREER, B. & MAKOWSKI, R. (1975). Gynecologic malignancies in immunosuppressed organ homograft recipients. *Obstet. Gynecol.*, **45**, 359.

RUDLINGER, R., SMITH, I.W., BUNNEY, M.H. & HUNTER, J.A.A. (1986). Human papilloma-virus infections in a group of renal transplant patients. *Br. J. Dermatol.*, **115**, 681.

SCHILSKY, R.L. & ERLICHMAN, C. (1982). Late complications of chemotherapy: Infertility and carcinogenesis. In *Pharmacological Principles of Cancer Treatment*, Chabner B. (ed.) p. 109. Saunders: Toronto.

SCHNEIDER, V., KAY, S. & LEE, H.M. (1983). Immunosuppression as a high risk factor in the development of condyloma acuminatum and squamous neoplasia of the cervix. *Acta Cytol.*, **27**, 220.

SHOKRI-TABIBZADEH, S., KOSS, L.G., MOLNAR, J. & ROMNEY, S. (1981). Association of human papillomavirus with neoplastic processes in the genital tract of four women with impaired immunity. *Gynecol. Oncol.*, **12**, S129.

SILLMAN, F.S., STANEK, A., SEDLIS, A. & 5 others (1984). The relationship between human papillomavirus and lower genital intraepithelial neoplasia in immunosuppressed women. *Am. J. Obstet. Gynecol.*, **150**, 300.

STREILEIN, J.W. (1978). Lymphocyte traffic, T-cell malignancies and the skin. *J. Invest. Dermatol.*, **71**, 167.

TESTER, W.J., KINSELLA, T.J., WALLER, B. & 2 others (1984). Second malignant neoplasms complicated Hodgkin's disease: the National Cancer Institute experience. *J. Clin. Oncol.*, **2**, 762.

WINKELSTEIN, W., SHILLITOE, E.J., BRAND, R. & JOHNSON, K.K. (1984). Further comments on cancer of the uterine cervix, smoking and herpes virus infection. *Am. J. Epidemiol.*, **119**, 1.